# Subacute thyroiditis after SARS‐CoV‐2 vaccination: A case report

**DOI:** 10.1002/ccr3.8678

**Published:** 2024-03-26

**Authors:** Ozra Akha, Mahdi Mazandarani, Soroush Azari, Niloofar Daneshfar, Kimia Rasouli, Keyvan Heydari, Golvash Tavakolian, Aref Hoseini

**Affiliations:** ^1^ Department of Internal Medicine, Diabetes Research Center, Faculty of Medicine Mazandaran University of Medical Sciences Sari Iran; ^2^ Endocrinology and Metabolism Research Center Tehran University of Medical Sciences Tehran Iran; ^3^ Student Research Committee, Faculty of Medicine Mazandaran University of Medical Sciences Sari Iran; ^4^ Kermanshah University of Medical Sciences Kermanshah Iran; ^5^ Gastrointestinal Cancer Research Center, Non‐Communicable Diseases Institute Mazandaran University of Medical Sciences Sari Iran

**Keywords:** case report, COVID‐19; SARS‐CoV‐2, subacute thyroiditis, vaccination

## Abstract

**Key Clinical Message:**

Subacute thyroiditis which is typically characterized by cervical pain and fever is caused by viral infection and is seen after SARS‐CoV‐2 vaccination. Here we report a post‐vaccination subacute thyroiditis after SARS‐CoV‐2 vaccination.

**Abstract:**

Subacute thyroiditis (SAT) is possibly caused by a viral infection and is typically characterized by cervical pain and fever. SAT associated with SARS‐CoV‐2 infection or SARS‐CoV‐2 vaccination has been reported, albeit in limited numbers. A 34‐year‐old woman was referred to our clinic with typical SAT symptoms. The diagnosis was confirmed through thyroid scintigraphy after receiving the SARS‐CoV‐2 vaccination, despite testing negative for COVID‐19 via RT‐PCR. There is a theoretical correlation between SARS‐CoV‐2 vaccination and SAT. Vaccination may have a direct or indirect impact on the thyroid, but further studies are required to confirm this relationship. A systematic review of the literature of similar cases was performed for comparison. Ultimately, the overall benefits of SARS‐CoV‐2 vaccination outweigh the potential adverse effects. Therefore, these types of reports should not divert attention from the actual reality.

## Graphical abstract

Thyroid scintigraphy of thyroid in the patient suspecting to SAT.

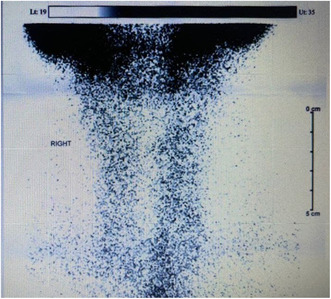

## 1 INTRODUCTION

Subacute thyroiditis (SAT) is a self‐limited inflammatory disorder that occurs in the thyroid gland.^1^ SAT is possibly caused by a viral infection and is typically characterized by anterior cervical pain radiating to the pharynx and jaw, along with fever.^2^ Laboratory studies reveal suppressed levels of TSH, elevated T3 and T4 levels, as well as increased WBC, CRP, and ESR levels. While anti‐thyroid peroxidase antibodies (Anti‐TPO) are typically found in painless SAT, they are usually absent or present in low titers in painful SAT.^3^ Thyroid scintigraphy evaluates the iodine‐123 iodide or ^99m^Tc‐pertechnetate uptake and distribution, which is expected to be markedly reduced in SAT.^4^, ^5^

SAT could play a role as a component of the COVID‐19 presentation with the signs mentioned above.^6^ In addition, SAT has been rarely reported after SARS‐CoV‐2 vaccination in some case reports.^1^, ^7^ It is suggested that SARS‐CoV‐2 vaccination may trigger autoimmune reactions of which SAT is the most prevalent example of it. Autoimmune/inflammatory syndrome induced by adjuvants (ASIA syndrome) is the other suggested reason behind SAT after vaccination due to the exposure to adjuvants. ASIA syndrome of Shoenfold's syndrome has been discussed first in 2011, as the reason behind several autoimmune conditions, which are induced following exposure to substances with adjuvant activity and should not be disregarded as a potential cause.^8^, ^9^, ^10^

We report a case of SAT following SARS‐CoV‐2 vaccination along with a literature review.

## 2 CASE HISTORY

A 34‐year‐old woman presented to our clinic with fatigue, sweating, anterior cervical pain, and fever lasting for 8 days. The patient mentioned receiving a SARS‐CoV‐2 vaccination 4 weeks ago, specifically the first dose of the BBIBP‐CorV (Sinopharm, Beijing CNBG (inactivated virus vaccine)) vaccine. She has a medical history of gastroesophageal reflux disease (GERD) and had a previous episode of COVID‐19 about 7 months ago, for which she did not require antiviral or corticosteroid therapy or hospitalization. She did not report any personal or familial history of thyroid diseases before, and she had no symptoms related to upper respiratory tract infections during these days. A physical examination was conducted, revealing an oral temperature of 38.5°C and tenderness in the anterior cervical‐thyroid anatomical area without any erythema.

## 3 METHODS

The nasopharyngeal swab polymerase chain reaction (PCR) test was negative for SARS‐CoV‐2. abratoryL studies, including CBC, inflammatory markers, thyroid function tests, and thyroid scintigraphy, shown in the table and figure below, were consistent with SAT probably associated with the preceding vaccine. Ultrasonography reported an upper limit of normal thyroid size with regular margins, decreased echogenicity, heterogeneous echotexture with hypoechoic areas, and normal vascularity in both lobes without nodules or cervical lymphadenopathy.

## 4 OUTCOME AND RESULTS

The treatment with 12.5 mg/day of prednisolone was initiated for the patient. During the follow‐up visit after 2 weeks, the primary symptoms had resolved, so the corticosteroid dose was gradually tapered.

## 5 DISCUSSION

### 5.1 SAT after vaccination

Several cases have been reported as subacute thyroiditis after COVID‐19 infection and SARS‐CoV‐2 vaccination which some of them are enlisted in Table 1 to facilitate any comparison. Now, subacute thyroiditis is a known complication or clinical manifestation of COVID‐19 with several reports from all over the world. SARS‐CoV‐2 could affect thyroid cells directly through ACE2 receptors (angiotensin‐converting enzyme) and the following inflammation.^11^

**TABLE 1:** Previous reported cases of post‐SARS‐CoV‐2 and COVID‐19‐associated SAT.

	Author	Age/gender	Type of vaccine	Para clinical Test for COVID‐19	Time passed from vaccination or COVID‐19 diagnosis	ignsS and symptoms	Thyroid tests	Inflammatory markers	Thyroid function in reassessment	Ultrasonography (US)	Thyroid scintigraphy	Treatment initiative and follow‐up
Post‐vaccination Case 1	Das et al.^24^	47/F	First dose of the ChAdOx1 nCoV‐19 (Astra Zeneca) vaccine	N/A	3 weeks	Fever/neck pain and tenderness/restlessness/difficult swallowing/weight loss	TSH ↓ T4 ↑ T3 ↑ Anti‐TPO (−) Anti‐TG (−) Anti‐TRAb (−)	–	N/A	ulky thyroid with hypoechoic nodulesB Without any cystic changes, calcification or increased vascularity	No uptake, consistent with thyroiditis	40 mg of propranolol every day/Symptoms resolved in 8 weeks
Post‐vaccination case 2	Chatzi et al.^1^	35/F	First dose of SARS‐CoV‐2 mRNA (Pfizer/BioNTech)	N/A	12 days	eck pain/fatigue/palpitationN	TSH ↓ FT4 ↑ Anti‐TPO (−) Anti‐TG (−) Anti‐TRAb (−)	WBC → ESR = 75 CRP = 498 (↑↑)	N/A	Increased gland dimensions with heterogeneous appearance and with hypoechogenic regions	Low uptake from the thyroid parenchyma with irregular gland margins	Prednisolone
Post‐vaccination case 3	Chatzi et al.	32/F	Second dose of SARS‐CoV‐2 mRNA (Pfizer/BioNTech)	N/A	4 days	Neck pain/fatigue	TSH ↓↓ FT4 → Anti‐TPO (−) Anti‐TG (−) Anti‐TRAb (−)	WBC → ESR = 40 CRP = 10 (↑)	N/A	Increased gland dimensions with heterogeneous appearance and hypoechogenic regions	Low uptake from the thyroid parenchyma with irregular gland margins	Prednisolone
Post‐vaccination case 4	Iremli et al.^25^	Prednisolone	Low Uptake from the thyroid parenchyma with irregular gland Margins	Increased Gland dimensions with heterogeneous appearance and Hypoechogenic regions	N/A	Neck pain/fatigue	TSH ↓↓ FT4 → Anti‐TPO (−) Anti‐TG (−) Anti‐TRAb (−)	WBC → ESR = 40 CRP = 10 (↑)	4 days	N/A	Second dose of SARS‐CoV‐2 mRNA (Pfizer/BioNTech)	Methylprednisolone started with 16 mg/day And propranolol 25 mg every 12 h
Post‐vaccination case 5	Iremli et al.	34/F	First dose of SARS‐CoV‐2 Vaccine (Vero Cell), Inactivated (CoronaVacR, Sinovac Life Sciences, Beijing)	Negative PCR	4 days	Anterior neck pain and tenderness/fatigue/and weight loss	TSH ↓ FT4 ↓ FT3 ↑↑ Anti‐TPO (−) Anti‐TG (−) Anti‐TRAb (−)	WBC → ESR = 19 CRP = 6(↑)	Thyrotoxicosis after 4 weeks	Bilateral focal hypoechoic areas with decreased blood flow	–	Methylprednisolone was started with 16 mg/day And propranolol 25 mg every 12 h
Post‐vaccination case 6	Iremli et al.	37/F	First dose of SARS‐CoV‐2 Vaccine (Vero Cell), Inactivated (CoronaVacR, Sinovac Life Sciences, Beijing)	Negative PCR	7 days	Neck pain and mild Tenderness on palpation over the right lobe of the thyroid Gland	TSH → FT4 → FT3 ↑ Anti‐TPO (−) Anti‐TG (−) Anti‐TRAb (−)	WBC → ESR = 25 CRP = 2.4 (→)	Thyrotoxicosis after 4 weeks	Bilateral hypoechoic areas with irregular borders and reduced blood flow in Doppler US	–	Followed up with no specific treatment and she was asymptomatic in Week 8
Post‐vaccination case 7	Saygılı et al.^26^	38/F	Second dose of SARS‐CoV‐2 Vaccine (Vero Cell), Inactivated (CoronaVacR, Sinovac Life Sciences, Beijing)	N/A	2 weeks	Swelling in the neck, pain, fatigue, loss of appetite and sweating	TSH ↓↓ FT4 ↑↑ FT3 ↑↑ Anti‐TPO (−) Anti‐TG (−)	WBC → ESR = 78 CRP = 8.76 (↑↑)	Hypothyroidism after about 1 month	Increased size of the right thyroid lobe, an irregularly demarcated hypoechoic area	–	Treatment started with 275 mg naproxen sodium and 20 mf propranolol, both of them twice a day After 14 days most of the complaints were resolved
Post‐vaccination case 8	Soltanpoor et al.^27^	34/F	First dose of COVAXIN (The Bharat Biotech COVID‐19 Vaccine)	Negative chest CT	5–7 days	Intermittent mild fever/Palpitation/anterior neck pain/mild thyroid enlargement and tenderness	TSH ↓ T4 ↑↑ T3 ↑	WBC → ESR = 60 CRP = 9.8 (→)	N/A	–	Moderate to severe decreased thyroid radiotracer uptake accompanied by high background activity, compatible with subacute thyroiditis	15 mg/day prednisolone, 20 mg propranolol, twice per day; The patient had a significant resolution of symptoms after 2 weeks
Post‐vaccination case 9	Siolos et al.^28^	51/F	First dose of the BNT162B2 SARS‐CoV‐2 (Pfizer‐BioNTech)	Negative PCR	4 days	Mild anterior neck pain/fever/nausea	TSH ↓ FT4 ↑ T3 → Anti‐TPO (−) Anti‐TG (−) Anti‐TRAb (−)	ESR = 103 CRP = 135 (↑↑)	Euthyroid after 8 weeks	–	Decreased uptake of 99mTc‐pertechnetate by the thyroid gland	16 mg/day of Methylprednisolone After 2 days fever and neck pain resolved
Post‐vaccination case 10	Siolos et al.	39/F	Did not mention the first or second dose/ChAdOx1‐S [recombinant] (AstraZeneca)	Negative PCR	–	No symptoms But abnormal thyroid function test	TSH ↓ FT4 ↑ T3 → Anti‐TPO (+) Anti‐TG (+) Anti‐TRAb (−)	ESR = 17 CRP = 1 (→)	Euthyroidism after 8 weeks	Profoundly hypoechoic left lobe with decreased blood flow	Decreased uptake and thyroid	Followed up with no specific treatment and laboratory tests were back to normal after 2 months
urrent Cstudy	–	34/F	With the first dose of BBIBP‐CorV (Sinopharm, Beijing CNBG)	Negative PCR	4 weeks	Fever/fatigue/sweating/anterior neck pain and tenderness	TSH ↓ T4 ↑ T3 ↑ Anti‐TPO N/A Anti‐TG N/A Anti‐TRAb N/A	ESR = 73 CRP = +3 D‐dimer ↑	N/A	Upper limit of normal size with regular margins, decreased echogenicity, heterogeneous echotexture with hypoechoic areas, normal vascularity in both lobes	Decreased uptake of 99mTc‐pertechnetate by the thyroid gland	12.5 mg/day of prednisolone Symptoms were resolved after 2 weeks
COVID Case 1	Davoodi et al.^6^	33/M	–	Positive PCR during SAT diagnosis	10	Fever/chills/sore throat/body ache/lethargy	TSH ↓ TT4 ↑ TT3 ↑ Anti‐TPO (−) Anti‐TRAb (−)	WBC ↑ ESR = 84 CRP = 37.9 (↑↑)	Euthyroidism after 7 weeks	A heterogeneous Thyroid gland with bilateral ill‐defined hypoechoic	N/A	Dexamethasone 4 mg every 8 h for 5 days and oral prednisone 25 mg daily with taper 7 weeks after he became Euthyroid
COVID Case 2	Chakraborty U et al.^29^	58/M	–	Positive PCR during SAT diagnosis	4	Fever/sore throat/neck tenderness/neck swelling/tachycardia	TSH ↓ TT4 ↑ TT3 ↑ Anti‐TPO (−) Anti‐TRAb (−)	WBC → ESR = 110 CRP = 16.6 (↑)	Hypothyroidism after 4 weeks	Increased vascularity of the thyroid gland and diffuse enlargement of the thyroid gland with hypoechogenicity and a solitary nodule in each lobe	Poor and patchy uptake of radiotracer in the thyroid gland with high circulating and background radioactivity	Initiate with 30 mg/day prednisolone then taper and 40 mg/day propranolol
COVID Case 3	Mari Des J. et al.^30^	47/F	–	Positive PCR during SAT diagnosis	14	Neck pain	TSH ↓↓ TT4 → TT3 → Anti‐TPO (−) Anti‐TRAb (−)	WBC → ESR = N/A CRP = 5 (↑)	Hypothyroidism after 8 weeks	A slightly enlarged right thyroid lobe, with ill‐defined hypoechogenicity and normal vascularity in both lobes	N/A	Celecoxib (dosage N/A) After 4 weeks resolve symptoms
COVID case 4	Khatri et al.^31^	41/F	–	Negative PCR during SAT diagnosis	14	Neck pain/neck swelling/fever/odynophagia/headache/chills/palpitations/fatigue/weight loss/hand Tremors	TSH ↓ T4 ↑ T3 ↑ Anti‐TPO (+) Anti‐TRAb (−)	WBC → ESR = 107 CRP = 36 (↑↑)	Euthyroid after about 6 weeks	A heterogeneous thyroid gland with bilateral patchy ill‐defined hypoechoic areas	N/A	Oral ibuprofen 600 mg every 6 h and prednisone 40 mg daily Complete symptoms resolution after 6 weeks from discharge
COVID case 5	Mattar SAM et al.^32^	34/M	–	Positive PCR during SAT diagnosis	9	Neck pain/tachycardia/diffuse asymmetric tender goiter/cervical lymphadenopathy	TSH ↓ FT4 ↑ FT3 ↑ Anti‐TPO (−) and Anti‐TRAb (−)	WBC ↑ ESR = N/A CRP = 122 (↑↑)	Euthyroid after 10 weeks	An enlarged thyroid gland with heterogeneous echotexture. Both lobes had hypoechoic areas with ill‐defined margins corresponding to the hard regions palpable. Color flow Doppler showed reduced blood flow in both lobes. A few cervical lymph nodes with normal morphology were seen	N/A	Initiate prednisolone at a dose of 20 mg. Atenolol at a dosage of 25 mg every morning
COVID case 6	Chong et al.^33^	37/M	–	Negative PCR during SAT diagnosis	30	Anterior neck pain with tenderness/fatigue/chills/palpitation/weight loss	TSH ↓ FT4 ↑ FT3 ↑ Anti‐TPO (−) and Anti‐TRAb (−)	WBC → ESR = 31 CRP = 14 (↑)	Hypothyroidism after 3 weeks	Thyroid gland echotexture is diffusely heterogeneous	N/A	Aspirin and propranolol
COVID case 7	Brancatella et al.^34^	18/F	–	Negative PCR during SAT diagnosis	19	Fever/fatigue/palpitations/anterior neck pain with tenderness	TSH ↓ FT4 ↑ FT3 ↑ Anti‐TPO (−) Anti‐TRAb (−) TgAb (+)	WBC ↑ ESR = 90 CRP = 6.9 (↑)	Euthyroid after 9 weeks	Multiple hypoechoic areas	N/A	Initiate with prednisone (25 mg/d) then tapered Resolve symptom within 1 week
COVID case 8	Mehmood MA et al.^35^	29/F	–	Negative PCR during SAT diagnosis	49	Fever/back pain/odynophagia/palpitation/weight loss/tachycardia/hand tremor	TSH ↓ FT4 ↑ FT3 ↑ Anti‐TPO (−) and Anti‐TRAb (−)	WBC → ESR = 84 CRP = 44 (↑)	Euthyroid after 10 weeks	Heterogeneously enlarged thyroid gland	N/A	initiate prednisone (20 mg) and atenolol (25 mg) daily then increase to 40 mg and 50 mg respectively Then tapered. Recovery during 10 weeks
COVID case 9	Asfuroglu Kalkan et al.^36^	41/F	–	Positive PCR during SAT diagnosis	0	Fever/neck pain and tender to palpitation	TSH ↓ FT4 ↑ FT3 ↑ Anti‐TPO (−) and Anti‐TRAb (−)	WBC ↑ ESR = 134 CRP = 101 (↑↑)	N/A	A relative diffuse decrease in vascularity and parenchyma was heterogeneous.	N/A	Prednisolone 16 mg daily and (drug for COVID‐19 such as hydroxychloroquine)
COVID case 10	Ruggeri RM et al.^4^	43/F	–	Negative PCR during SAT diagnosis	45	Pain and tenderness of neck/fatigue/tremors/palpitations/cervical and submandibular lymphadenopathy	TSH ↓ FT4 ↑ FT3 ↑ Anti‐TPO (−) Anti‐TRAb (−)	WBC → ESR = 60 CRP = 8.8 (↑)	Euthyroid after 4 weeks	A diffusely Enlarged and hypoechogenic thyroid gland	Markedly reduced 99mTc uptake in the gland	Oral prednisone (25 mg/day as the starting dose, gradually tapered) resolution symptoms during 4 weeks

Abbreviations: TSH: ↓ 0.01–0.2 μIU/mL, ↓↓ <0.01 μIU/mL. T3, FT3, T4, FT4, WBC, CRP and etc.: → within normal; ↑, increased; ↓, decreased; ↑↑, 1.5 times more than normal upper limit; ↓↓, 1.5 times lower than normal lower limit; TSH, thyroid‐stimulating hormone; (F)T3, (free) triiodothyronine; (F)T4, thyroxine; anti‐TPO, thyroid peroxidase Antibody; WBC, white blood cell; CRP, C‐reactive protein; ESR, erythrocyte sedimentation rate; Anti‐Tg, anti‐thyroglobulin antibody; TRAB, thyroid receptor antibodies; N/A, not assessed.

Thyroid disorders following viral infections or vaccinations have been discussed in the past decades. After the COVID‐19 pandemic and the production of vaccines with different structures and their injection, thyroid‐related disorders were seen.^12^ Studies have shown that these disorders have occurred with the injection of almost all types of COVID‐19 vaccines, although the highest rate has occurred with mRNA‐based vaccines, followed by viral vectors, and less in inactive vaccines. These disorders include subacute thyroiditis, Graves' disease, focal painful thyroiditis, and finally silent thyroiditis.^13^ Of course, in some cases, concurrent Graves' disease and SAT have been reported, too.^14^

Subacute thyroiditis, which is also referred to as granulomatous or de Quervain's thyroiditis, is a self‐limiting autoimmune condition that occurs in response to a viral infection or the resulting inflammatory state. It usually presents with fever, neck pain, and palpitations; however, in some cases, patients do not develop any significant symptoms.^12^ Typical SAT has three sequential phases, which include thyrotoxicosis, hypothyroidism, and euthyroidism. It is believed that this condition occurs after a recent viral infection in genetically predisposed patients, and the relationship between numerous HLAs (such as HLA‐Bw35, HLA‐B67, HLA‐B35, HLA‐DRB1*08*, *HLA‐DRB1*01, HLA‐B*18:01*, *HLA‐DRB1*01, and HLA‐C*04:01) and SAT has been established.^15^, ^16^

SAT mostly affects middle‐aged women, and as we mentioned earlier, it is common after receiving viral vaccinations. Jafarzadeh et al.^13^ gathered data on 50 SAT cases, of which 62% occurred following mRNA‐based vaccines, 24% with inactivated vaccines, and 12% with viral vector vaccines. In one patient, there was no data about the type of vaccine. They also stated that most patients were female, with a mean age of 39.5 years, and 58% of patients experienced SAT after the first vaccine injection. Similar to our study, the patient was a 34‐year‐old woman who had a history of receiving the first dose of an inactivated viral vaccine before developing SAT.

**FIGURE 1:**
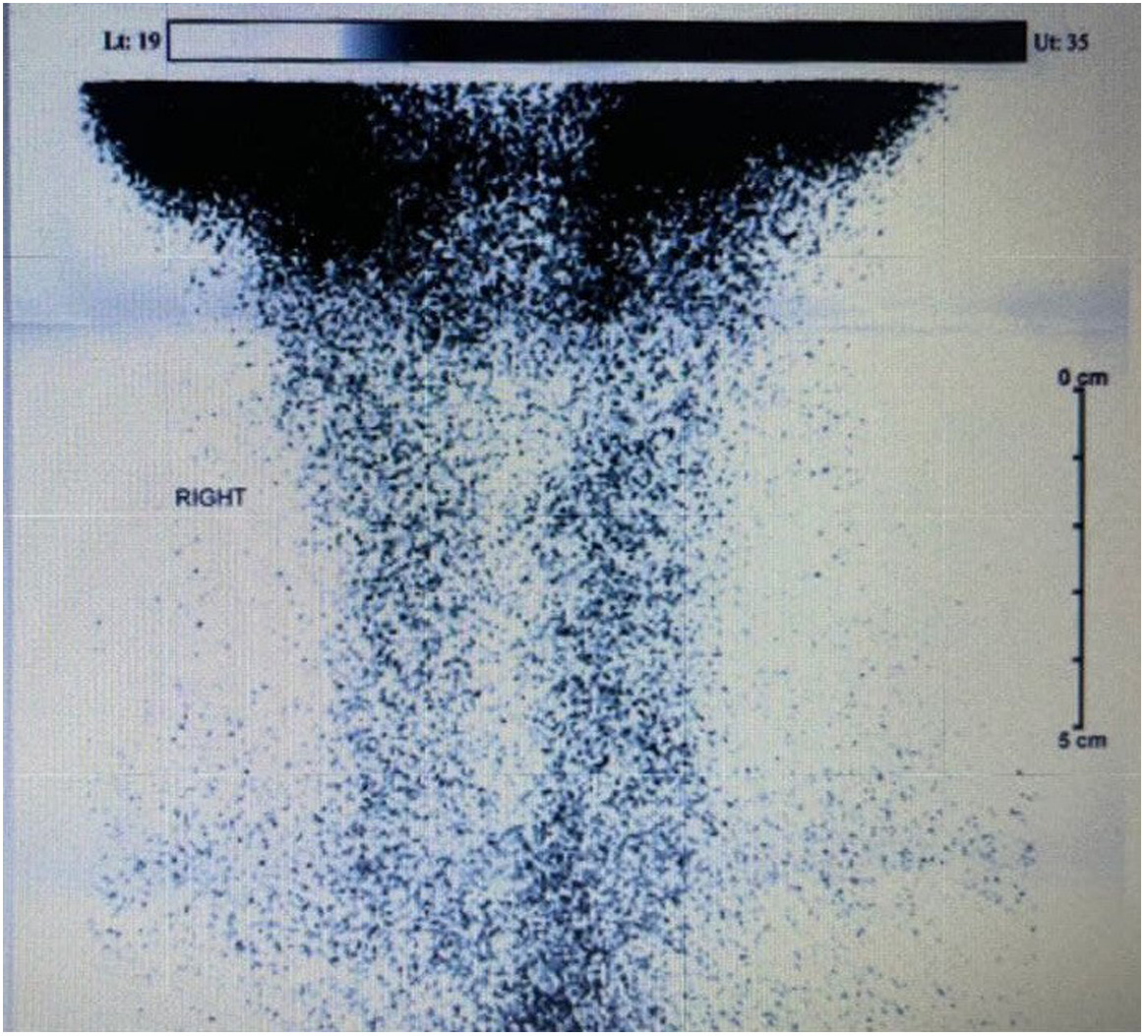
Scintigraphy (^99m^Tc scan) demonstrates poor radiotracer uptake of both lobes with increased background and salivary gland uptake consistent with SAT.

The diagnosis of SAT is based on laboratory findings, ultrasonography (US), and scintigraphy features. Elevated erythrocyte sedimentation rate (ESR) and C‐reactive protein (CRP), as well as abnormal thyroid function tests, are seen in patients. In the US, hypoechoic and heterogeneous pieces with obscure borders and feeble vascularization are characteristic features. In line with our study, as shown in Table 2, our patient had high levels of ESR, CRP, T3, T4, and low TSH. On US evaluation, there was an upper limit‐sized thyroid with regular margins and decreased echogenicity, heterogeneous echotexture with hypoechoic areas.^17^ The thyroid scintigraphy usually shows a low or absent uptake, similar to our case, Figure 1, Scintigraphy (99mTc scan) demonstrates poor radiotracer uptake of both lobes with increased background and salivary gland uptake consistent with SAT.^18^

**TABLE 2:** Laboratory results of the patient on admission date and follow‐up.

Laboratory tests	Admission	After 8 weeks	Reference range
ESR	73	5	0–20
CRP	+3	Neg	Neg
D‐dimer	727	Neg	Neg: <500
TSH	0.1	4.64	0.4–5.5
T4	16.8	8.5	4.5–12.5
T3	489	–	84–172
WBC	9100	9300	4400–11,300
Lymphocytosis	1700	3350	800–4000
Hgb	9.7	12.7	12.5–15.3
MCV	74.9	76.4	80–96.1
MCH	23.8	25.4	27.7–35
PLT	302,000	250,000	150,000–450,000

Abbreviations: CRP, C‐reactive protein; ESR, elevated erythrocyte sedimentation rate; Hgb, hemoglobin; MCH, mean corpuscular hemoglobin; MCV, mean corpuscular volume; PLT, platelet; TSH, thyroid‐stimulating hormone; WBC, white blood cell.

Steroids and non‐steroidal anti‐inflammatory drugs (NSAIDs) are mostly effective in treating the symptoms and normalizing the laboratory markers of SAT.^19^ In our study, after diagnosing SAT, we prescribed prednisolone (12.5 mg daily) for the patient, and in a 2‐week follow‐up, her condition improved.

### 5.2 A relation behind the vaccine injection and thyroid diseases

As stated in the studies, thyroid cells produce the SARS‐CoV‐2 receptor called angiotensin‐converting enzyme 2 (ACE2) and the transmembrane protease serine 2 (TMPRSS2). Therefore, SARS‐CoV‐2 can assault thyroid tissue, leading to thyroid dysfunction during and after COVID‐19 infection. Antibodies against SARS‐CoV‐2S protein have been reported to react with thyroid peroxidase (TPO) and can directly bind to ACE2‐expressing thyroid cells. These antibodies may play a role in initiating autoimmunity through molecular mimicry in susceptible individuals. Additionally, studies have reported a positive relationship between clinical severity associated with COVID‐19 and thyroid dysfunction.^13^, ^17^, ^20^

Vaccines, like infections, may play a role in the development of autoimmune conditions through different mechanisms, such as molecular mimicry, epitope spreading, polyclonal activation, and the presentation of enigmatic antigenic determinants. Molecular mimicry between vaccine antigens and thyroid proteins can trigger an autoimmune response. Several factors, such as tissue damage, prolonged inflammatory response, and genetic background, can also cause autoimmune diseases.^13^

Based on the above explanations, patients infected with SARS‐CoV‐2 or vaccinated may be at risk for thyroid dysfunction, especially those with a prolonged inflammatory response and a genetic background.

However, it was observed by Clarke et al.^21^ that thyroid and adrenal function were found to be preserved ≥3 months after the onset of COVID‐19. Although a considerable number of individuals had chronic fatigue, changes in thyroid or adrenal function could not explain their symptoms. In addition, Goyal et al.^22^ conducted longitudinal cohort research in which subjects examined at a short time (<1 year) after a primarily mild and asymptomatic SARS‐CoV‐2 infection did not show signs of thyroid autoimmune or dysfunction progressing. In our opinion, the risk of subacute thyroiditis due to SARS‐CoV‐2 infection or vaccination is different for each person and occurs rarely. However, according to the history of autoimmune disorders and the specific conditions of each person, therapeutic and preventive protocols should be undertaken in susceptible people.

The cases presented, including those outlined in Table 1, occurred following SARS‐CoV‐2 vaccination. This occurrence is considered coincidental, and the theories discussed represent potential pathways rather than established causation. Further investigations are essential to provide a more comprehensive understanding of this potential correlation.

It is important to emphasize that SARS‐CoV‐2 vaccination has resulted in a substantial reduction in mortality and disease severity among the general population. The occasionally reported adverse events should not undermine the significant benefits associated with vaccination.^23^

## 6 CONCLUSION

Despite numerous studies, it is still impossible to definitively determine the effect of being infected with COVID‐19 or receiving a SARS‐CoV‐2 vaccine on the occurrence of autoimmune thyroid disease. This issue has become more complicated, especially considering several factors, including the different types of vaccines administered to different individuals. We hereby present a documented instance of SAT following SARS‐CoV‐2 vaccination, accompanied by a thorough a brief review concerning COVID‐associated and post‐vaccination cases of SAT. We have organized the relevant data in Table 1 to facilitate comparisons.

## AUTHOR CONTRIBUTIONS

**Ozra Akha:** Conceptualization; investigation; validation; writing – original draft. **Mahdi Mazandarani:** Investigation; writing – original draft; writing – review and editing. **Soroush Azari:** Data curation; writing – original draft. **Niloofar Daneshfar:** Conceptualization; project administration; writing – original draft; writing – review and editing. **Kimia Rasouli:** Investigation; writing – review and editing. **Keyvan Heydari:** Conceptualization; investigation; writing – original draft; writing – review and editing. **Golvash Tavakolian:** Writing – original draft. **Aref Hoseini:** Conceptualization; data curation; investigation; project administration; resources; supervision; validation; writing – original draft; writing – review and editing.

## FUNDING INFORMATION

This research did not receive any specific grant from funding agencies in the public, commercial, or not‐for‐profit sectors.

## CONFLICT OF INTEREST STATEMENT

The authors declare no competing interest relevant to the contents of this article.

## ETHICS STATEMENT

The study was approved by the Ethics Committee of Mazandaran University of Medical Sciences.

## CONSENT

Written informed consent was obtained from the patient for publication of this case report and any accompanying images. A copy of the written consent is available for review by the Editor‐in‐Chief of this journal.

## Data Availability

All data generated or analyzed during this study are included in this published article (and its additional information files).
